# WT1 Cancer Vaccine in Advanced Pancreatic Cancer: A Systematic Review

**DOI:** 10.7759/cureus.56934

**Published:** 2024-03-26

**Authors:** Kalyan Naik Gugulothu, Pampatti Anvesh Sai, Sonika Suraparaju, Sai Prasad Karuturi, Ganesh Pendli, Ravi babu Kamma, Kethana Nimmagadda, Alekhya Modepalli, Mahesh Mamilla, Shambhavi Vashist

**Affiliations:** 1 Internal Medicine, Sri Venkateswara Medical College, Tirupati, IND; 2 Internal Medicine, Katuri Medical College and Hospital, Guntur, IND; 3 Internal Medicine, Sri Padmavathi Medical College for Women, Tirupati, IND; 4 Internal Medicine, Sri Venkata Sai (SVS) Medical College, Mahabubnagar, IND; 5 Internal Medicine, PES Institute of Medical Sciences and Research, Kuppam, IND; 6 Internal Medicine, AMA School of Medicine, Makati, PHL; 7 Internal Medicine, NC Medical College and Hospital, Panipat, IND

**Keywords:** gemcitabine, pancreatic cancer treatment, wt1, cancer vaccines, advanced pancreatic cancer

## Abstract

Advanced pancreatic cancer is one of the prominent contributors to cancer-related mortality globally. Chemotherapy, especially gemcitabine, is generally used for the treatment of advanced pancreatic cancer. Despite the treatment, the fatality rate for advanced pancreatic cancer is alarmingly high. Thus, the dire need for better treatment alternatives has drawn focus to cancer vaccinations. The Wilms tumor gene (WT1), typically associated with Wilms tumor, is found to be excessively expressed in some cancers, such as pancreatic cancer. This characteristic feature is harvested to develop cancer vaccines against WT1. This review aims to systematically summarize the clinical trials investigating the efficacy and safety of WT1 vaccines in patients with advanced pancreatic cancer. An extensive literature search was conducted on databases Medline, Web of Science, ScienceDirect, and Google Scholar using the keywords "Advanced pancreatic cancer," "Cancer vaccines," "WT1 vaccines," and "Pulsed DC vaccines," and the results were exclusively studied to construct this review. WT1 vaccines work by introducing peptides from the WT1 protein to trigger an immune response involving cytotoxic T lymphocytes via antigen-presenting cells. Upon activation, these lymphocytes induce apoptosis in cancer cells by specifically targeting those with increased WT1 levels. WT1 vaccinations, which are usually given in addition to chemotherapy, have demonstrated clinically positive results and minimal side effects. However, there are several challenges to their widespread use, such as the immunosuppressive nature of tumors and heterogeneity in expression. Despite these limitations, the risk-benefit profile of cancer vaccines is encouraging, especially for the WT1 vaccine in the treatment of advanced pancreatic cancer. Considering the fledgling status of their development, large multicentric, variables-matched, extensive analysis across diverse demographics is considered essential.

## Introduction and background

Pancreatic cancer remains a highly lethal disease. Even with continuous improvements in diagnosis and treatment, a high number of deaths are noted, especially if the disease is advanced [1,2]. The mainstay of care for unresectable, locally progressed, and metastatic pancreatic ductal adenocarcinoma (PDA) has been gemcitabine (GEM) monotherapy since 1997. However, GEM has a one-year overall survival (OS) rate of less than 20% and a median OS of roughly six months [3].

In an attempt to increase the efficacy of treating advanced pancreatic cancer (AdPC), GEM in combination with other medicines has been investigated. A few randomized trials have demonstrated the benefits of combination therapy over GEM alone, despite the fact that there have been several non-fruitful studies [4]. Innovative therapy approaches are therefore desperately needed to improve prognosis. Work on cancer immunotherapies, especially peptide-based cancer vaccines that target tumor-associated antigens (TAAs), is one of the promising strategies now under investigation. These vaccines aim to promote the growth of cytotoxic T lymphocytes (CTLs) that are specific to TAAs in order to eradicate cancer cells [4]. While cancer immunotherapy can sensitize tumor cells to subsequent chemotherapeutic drugs, the combination of cancer vaccines and chemotherapy has the advantage of making tumor cells receptive to CTL responses. As a result, it is anticipated that some chemotherapeutic drugs and cancer vaccines would work in concert to improve patient outcomes and quality of life in advanced cancer patients [5,6].

Wilms' tumor gene 1 (WT1), which was once thought to be a tumor suppressor gene causing Wilms' tumor, is one of the most promising TAAs that has been identified as a target for cancer immunotherapy [7]. In the 2009 National Cancer Institute-sponsored ‘Cancer Antigen Prioritization Project’, WT1 was ranked as the top antigen for priority research [8]. WT1 has been shown to have oncogenic activities in cancer, including growth promotion, differentiation inhibition, resistance to cell death, and tumor angiogenesis facilitation, despite its original characterization [9]. Interestingly, PDA and other malignancies have overexpressed wild-type WT1, which acts as a poor prognostic indicator [10,11]. Consequently, studies have been initiated to investigate the possibility of using a WT1 cancer vaccine to treat PDA. The present work aims to conduct a systematic review of clinical trials on the use of a WT1-based strategy for treating AdPC.

## Review

Research design and outcomes

Literature Search Strategy

**Figure 1:**
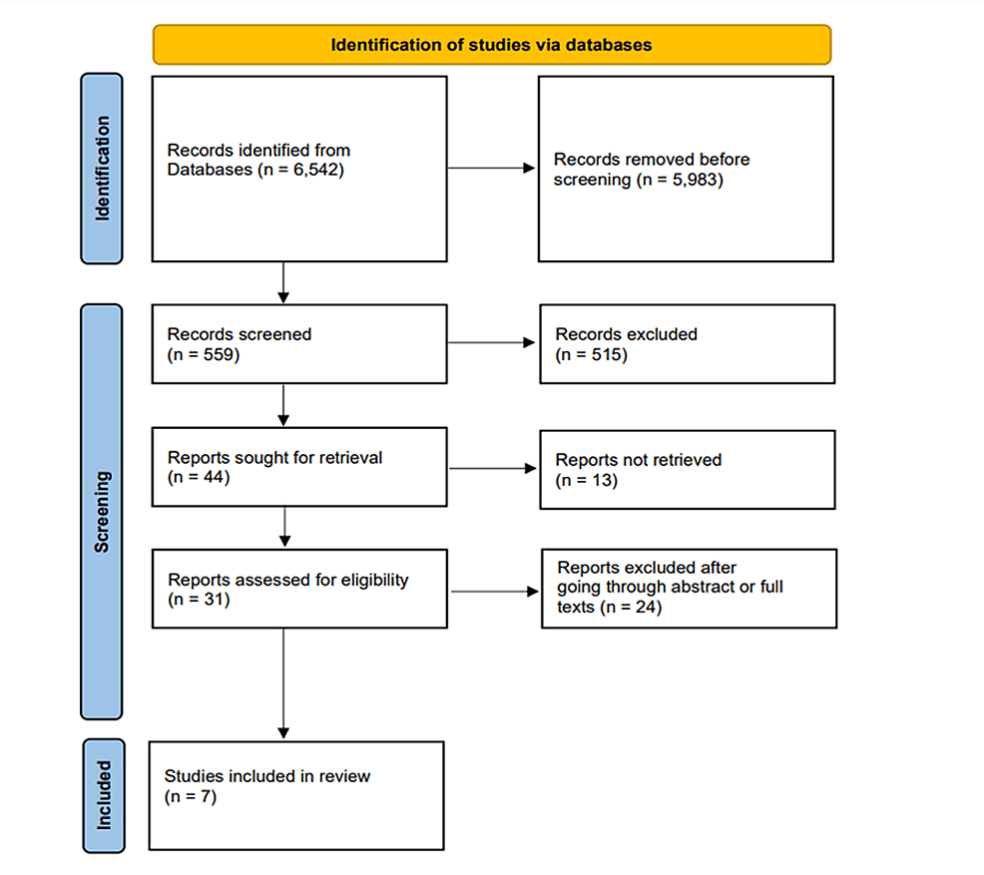
Preferred Reporting Items for Systematic Reviews and Meta-Analyses (PRISMA) 2020 Flow Diagram

Figure 1 describes the search approach and studies selected for this systematic review using the Preferred Reporting Items for Systematic Reviews and Meta-Analyses (PRISMA) 2020 flow diagram [12]. Databases, Medline, Web of Science, ScienceDirect, and Google Scholar, were selected for a thorough search of all original research without time restrictions by a selected set of key terms, "Advanced pancreatic cancer," "Cancer vaccines," "WT1 vaccines," and "Pulsed DC vaccines."

Criteria for Eligibility

We employed a strategic criterion to reduce the number of publications, as the search generated a large pool of papers. Human clinical trials written in the English language up until October 1, 2023, the last day of our data search, were included in the inclusion criteria. Exclusion criteria included in vitro studies, animal studies, review articles, conference abstracts, letters to the editor, case series, and case reports. Studies published in languages other than English were also excluded.

Data Extraction and Analysis

We discovered 6,542 articles from our initial search using the key terms. Following the application of exclusion and inclusion criteria, 5,923 articles could be eliminated. Because of duplicates or issues with their titles, we chose not to use 960 of the remaining 1,519 papers. Of the remaining 559 articles, 515 were excluded as they did not meet the standards, 13 were not retrieved, and 24 were excluded after going through abstracts or full texts. The Newcastle-Ottawa Scale for clinical trials served as a quality assessment instrument to determine the eligibility of the papers we chose [13]. The Prediction Model Risk of Bias Assessment Tool (PROBAST) was used to evaluate the bias risk and applicability of the chosen studies [14]. Any discrepancies were resolved through discussion and consensus.

Results

General information, study group particulars, treatment details, objectives, and outcomes of the included research works were evaluated and tabulated in Table 1 and Table 2. After a thorough analysis of the studies, a note was added below the tables.

**Table 1:** Information About Clinical Trials WT1: Wilms’ tumor gene 1, Gemcitabine: GEM, University Hospital Medical Information Network: UMIN, DC: dendritic cells, MHC: major histocompatibility complex, MUC1: mucin, OS: overall survival.

S. No.	Author	Year of publication	Country of study	Phase of the trial	Study particulars	Intervention particulars	Objective of the study
1	Kaida et al. [15]	2011	Japan	Phase I	Dose escalation trail registered at and approved by the National Cancer Center of Japan	WT1 vaccine and GEM combination therapy	Assessment of the WT1 vaccine's approximate immunologic dosage, safety profile, and toxicity
2	Nishida et al. [16]	2014	Japan	Phase I	Registered with the UMIN. Trial registration ID: UMIN-000001187	WT1 peptide-based cancer vaccine combined with GEM	Investigate the feasibility and efficacy of this combination therapy and the immunologic response to WT1 peptide
3	Koido et al. [17]	2014	Japan	Phase I	Registered with the UMIN. Trial registration ID: UMIN-000004063	3 groups - each group receiving GEM + one of the three restricted WT1 vaccines, MHC class I restricted (WT/DC/I) or MHC class II-restricted (WT/DC/II) or both (WT1/DC/I+II)	Analyze the clinical responses and safety profile and learn about vaccine-particular immune responses
4	Mayanagi et al. [18]	2015	Japan	Phase I	Registered with the UMIN. Trial registration ID: UMIN-000004855	WT1 peptide-pulsed DC vaccine combined with GEM	Assess the safety and OS of this combination
5	Nishida et al. [19]	2018	Japan	Phase II	Registered with the UMIN. Trial registration ID: UMIN-000005248	WT1+GEM compared it with GEM alone	Learn the efficacy of combined therapy compared to monotherapy
6	Yanagisawa et al. [20]	2018	Japan	Phase I	Registered at and approved by Shinshu University School of Medicine (approval numbers 1123 and 1199)	WT1-pulsed DC Vaccine Combined with Chemotherapy (S-1 alone or S-1+GEM	Assess the safety and outcomes of combined therapy
7	Nagai et al. [21]	2020	Japan	Phase I/IIa	Registered with the UMIN trial registry	WT1 peptide and MUC1 pulsed DC (WT1/MUC1-DC) vaccine and concurrent adjuvant chemotherapy	Assessment of the safety profile was the main objective of this study

**Table 2:** Findings From Clinical Trials HLA: human leukocyte antigen, PDA: pancreatic ductal adenocarcinoma, WT1: Wilms’ tumor gene 1, GEM: gemcitabine, MUC1: mucin, DC: dendritic cells, IV: intravenously, ID: intradermally, MSL: median survival time, DTH: delayed-type hypersensitivity, CTLs: cytotoxic T-lymphocytes, ICC: intrahepatic cholangiocarcinoma, OS: overall survival, PFS: progression-free survival, RFS: relapse-free survival.

S. No.	Authors	Study group information	Size of the study	Treatment details	Results	Survival rate	Notes
1	Kaida et al. [15]	HLA-A 02:01, HLA-A 02:06, and/or HLA-A 24:02-positive patients with inoperable advanced PDA or biliary tract cancer who had not previously been treated with GEM were eligible for this study	25, but only 9 had advanced PDA (8 had gallbladder cancer, 4 had intrahepatic, and 4 had extrahepatic bile duct cancer)	WT1+GEM every 28 days (over 2 months) as outlined: GEM – IV route, 1000 mg/m^2^ on 1st, 8th, and 15th days, followed by a 7-day break. WT1 – ID route, 0.1 mL on 8th, and 22nd days	# Negative effects were equivalent to those caused by GEM alone. The safety of WT1 vaccination and GEM combined therapy was validated in this investigation. However, a lack of obvious objective clinical efficacy is observed. # Disease control rate was 89% for pancreatic cancer at 2 months	MST for pancreatic cancer was 259 days, while the MST for cancer of the biliary tree was 288 days. 37 weeks was the median follow-up time frame	Two patients had positive results from a DTH test following immunization, and 59% of the patients had WT1-specific T cells identified by tetramer assay in peptide-stimulated culture
2	Nishida et al. [16]	Patients with pathologically or cytologically confirmed, measurable, locally advanced, or metastatic PDA or with recurrent disease were recruited for this. Another major criterion put for eligibility is HLA-A 24:02 positivity	32	GEM (1000 mg/m^2^), given IV on days 1, 8, and 15 of a 28-day cycle. WT1 vaccine was administered ID at six different sites (bilateral upper arms, lower abdomen, and femoral regions) on days 1 and 15 of a 28-day cycle with initial treatment protocol planned for two courses	# Combined therapy was well tolerated, as seen by the similar frequency of higher grade (grade III/IV) adverse effects compared to GEM alone. # In six of the 30 evaluable patients, objective response rate stood at 20%	This study reported an MST of 8.1 months while the 1-year survival rate was 29%	A statistically significant connection was noted between prolonged survival and positive DTH. Prior to and following treatment, there was an increased frequency of WT1-specific CTLs with a memory phenotype in prolonged survivors. This could be a useful prognostic marker for survival in WT1+GME therapy
3	Koido et al. [17]	Patients with pathologically or cytologically confirmed, measurable, metastatic pancreatic or biliary tract adenocarcinoma or with recurrent disease and positive for HLA-A 02:01, HLA-A 02:06, HLA-A 24:02, DRB1 04:05, DRB1 08:03, DRB1 15:01, DRB1 15:02, DPB1 05:01, or DPB1 09:01. were selected for this study	11: 10 patients with stage IV PDA and 1 patient with ICC	One cycle of only GEM, IV route, 1,000 mg/m^2^ on 1st, 2nd, and 15th days in a 4-week cycle, followed by GEM+Vaccine. GEM dosing and timing are similar to the first cycle. The vaccine is given via the ID route twice a week at six different sites. The initial treatment protocol was planned as three courses. 2 PDA patients and 1 ICC patient were given WT1/DC/I. 1 patient with PDA was treated with WT1/DC/II. The remaining 7 patients with PDA were given WT1/DC/I+II	# The paired treatment was received well by patients. After being treated with WT1/DC/I+II, there was a considerable increase in WT1-specific IFNγ-producing CD4(+) T cells. # All patients experienced reversible, grade 1 skin reactions at the site of vaccination	# When compared to the negative control patients, the OS and PFS of the WT1-specific DTH-positive patients were noticeably better. Specifically, the median OS for all three PDA patients who experienced strong DTH reactions was 717 days. # Compared to patients receiving the WT1/DC/I or WT1/DC/II vaccines, individuals with PDA who received the WT1/DC/I+II vaccine had a significantly longer MST (P = 0.036) and median PFS (P = 0.010)	After receiving the WT1/DC/I+II vaccination, patients were divided into two groups based on OS: Non-super-responders (has OS < 1 year) and super-responders (has OS ≥ 1 year). After seven or eight vaccinations, the study found three super-responders but was unable to discern a difference in the percentage of WT1-CTLs in the overall CD8+ T-cell populations between these two groups
4	Mayanagi et al. [18]	HLA-A 24:02 positive patients with advanced PDA	10	WT1 peptide-pulsed DC vaccine (dose of 10^7^) was injected ID in close proximity to the axillary or inguinal lymph nodes on days 8 and 22. Gem (1000 mg/m^2^) was administered every 4 weeks by IV drip infusion for 30 min on days 1, 8, and 15. A total of three cycles of DC vaccination were planned	# The hematological side effects of GEM therapy did not seem to be exacerbated by the vaccine. # The disease control rate was 60% for this study. # Despite inducing a WT1-specific immune response, patients with liver metastases and high levels of inflammatory markers such as C-reactive protein and interleukin-8 had a poor prognosis	The median OS rate was 243 days. Individuals with stable disease had a substantially higher post-treatment survival rate than individuals with progressing disease (P = 0.016)​	50% of patients finished the protocol, while the remaining 50% stopped, due to either a serious adverse event, such as interstitial pneumonia linked to GEM treatment, or fast disease progression
5	Nishida et al. [19]	HLA-A 02:01 or HLA-A 24:02 positive patients with histologically or cytologically confirmed locally advanced or metastatic PDA Subjects were randomly assigned to receive GEM+WT1 or GEM	91 enrolled patients, but 85 were evaluated (GEM+WT1: n = 42; GEM: n = 43)	One group of patients was given only GEM, IV route, 1,000 mg/m^2^ on the 1st, 8th, and 15th days in a four-week cycle. The second group received a combination therapy. WT1 peptide vaccination was given to patients in the ID route at six different sites on the 1st and 15th day of a four-week cycle. GEM dose and timing remain the same as of the first group	# Two groups' baseline quality of life scores (assessed by the FACT-G scale) were comparable. The GEM+WT1 group's overall score rose following therapy. In the fourth course, the GEM+WT1 group's mean total score was 80.2, higher than the GEM group's mean total score of 70.4, but P = 0.063, which denotes a statistically insignificant difference. # There were no documented treatment-related deaths, and there were no appreciable variations in the frequency of any adverse events between the two groups. # 52.4% and 37.2% in the GEM+WT1 group and GEM group (P = 0.194) respectively was the reported disease control rate	# The median OS of the GEM+WT1 group and GEM group was 9.6 and 8.9 months respectively (HR, 0.82; 90% CI, 0.57–1.18, P = 0.363). The difference was not statistically significant. # Nevertheless, the difference in median PFS is statistically significant, 5.2 and 3.3 months respectively (HR, 0.66; 90% CI, 0.44–0.98, P = 0.084)	# A correlation was found between the activation of immune responses specific to WT1 and clinical outcomes. # A rise in WT1-CTLs brought on by the WT1 vaccination was linked to DTH-positive
6	Yanagisawa et al. [20]	Patients with PDA who underwent resection after initial diagnosis and then received chemotherapy. Excluded patients who received chemotherapy before surgery	08	WT1-pulsed DC vaccine was given ID and bilaterally near the axillary region and groin. As a course, it was administered seven times every 2 weeks. Regarding adjuvant chemotherapy-7 patients received S-1 alone and one received S-1+GEM after surgery	# Most of the adverse events reported were in lower grades (grade 1/2). # WT1-specific CTLs were detected in seven patients, and WT1 and HLA-I antigens were positive in all 34 cases	OS at 2 years after the operation was 62.5±17.1% (95% confidence interval=22.9-86.1%)	Five patients' tetramer assay findings before and after WT1-DC immunization did not change significantly (p=0.1250). However, after comparing the Enzyme-linked immunosorbent spot (ELISPOT) assay findings for every patient, a significant increase in score was seen (p=0.0156)
7	Nagai et al. [21]	Subjects who had HLA class I genotypes compatible with restriction of the WT1 peptide and diagnosed with PDA and underwent respective operations after diagnosis	10	1×10^7^ WT1/MUC1-DCs were injected ID at four positions in the axilla and groin regions on each side seven times in 2-week intervals. OK-432 was administered subcutaneously in each axilla (0.5 mL each) to activate DC functions. Concurrent adjuvant chemotherapy included the S-1 (orally at a daily dose of 80-120 mg for 14-28 days with 1-2 weeks rest repeated every 3-6 weeks) in 8 patients, GEM (100 mg/m^2^ on day 1 and repeated every 3 weeks with 1-2 weeks rest) in 1 patient. One patient refused adjuvant chemotherapy	Erythema, or skin reaction at the injection site of the DC vaccination, was the most frequent adverse event of any grade, followed by fever. These adverse events were brief and controllable with symptomatic therapy, thus there was no need to postpone the prescribed course of care. Overall, a solid safety profile was established	The estimated OS and RFS at 3-years were 77.8% (95%CI=0.37-0.94) and 35.0% (95%CI=0.09-0.64), and those at 5-year were 19.4% (95%CI=0.01-0.55) and 23.3% (95%CI=0.04-0.53), respectively. The OS and RFS were 18.5-72.8 months (median 46.4 months) and 12.5-72.8 months (median 17.7 months), respectively	# Higher infiltration of CD3/CD4/CD8 cells in tumor tissues may be related to the production of WT1-specific CTLs following DC immunization, according to immunohistochemical studies. # This work is the first to describe the viability and safety of DC-based tumor-specific immunization for PDA as an adjuvant setting following surgical tumor excision, employing a combination of WT1 peptide and MUC1-DC

According to our analysis of clinical trials, WT1 cancer vaccines are safe to use and do not worsen side effects when used in conjunction with chemotherapy, which usually involves the drug GEM. Adverse effects attributed to cancer vaccinations include injection site skin reactions and low-grade fever (grade 1 most often, grade 2 occasionally), all of which are non-serious and tend to resolve during the course of treatment. Although WT1 vaccinations were associated with a higher median survival rate and longer survival duration, some of the studies showed no statistically significant difference (p>0.05). It is important to note that all of the studies in this review are Phase I/II trials, even if the results of the clinical trials thus far have been positive. As a consequence, as the phase goes on, the results may show variability. Therefore, it is advised to continue close monitoring.

It is also important to note that all the trials that were examined were carried out in Japan, and extra care should be taken when extrapolating these results to other demographics. One noteworthy finding is that human leukocyte antigen (HLA) criteria were included in the patient selection procedure in all seven of the trials included in this study. This was made necessary by the fact that the HLA-A*24:02 allele is present in about 60% of the Japanese population [22]. The HLA status and other pertinent features of the populations being examined must match for the results of this study to be extrapolated. The majority of participants in all the investigations had positive delayed-type hypersensitivity (DTH) test results. This allows us to conclude that WT1 vaccinations elicit a substantial immunological response in the host body, while individual participants may show varied clinical changes significantly.

Shortcomings and Proposed Directions for Future Clinical Studies

As was already indicated, the demographics of the included subjects represent a major limitation in these investigations. A lack of a multicentric approach in certain studies may introduce bias in the selection process. Another notable drawback is the population size, albeit in the context of Phase 1 or 2 trials. It is worth noting that the predominant focus of most studies centers around GEM chemotherapy. Although GEM is still the mainstay of treatment, it is important to recognize that other commonly used chemotherapeutic drugs exist in this field, indicating a possible direction for future research.

In order to overcome these constraints, we propose the use of multicentric studies that include a range of demographic characteristics in addition to thoughtful consideration of an increased sample size. Moreover, we suggest that investigations in the future encompass a wider range of chemotherapeutic drugs that are frequently employed in the therapeutic context. This all-encompassing strategy seeks to reduce participant selection biases, improve generalizability, and offer a more detailed knowledge of the interplay between different chemotherapy regimens and WT1 cancer vaccines.

Discussion

Pancreatic Cancer

AdPC poses a significant challenge within the field of oncology due to poor prognosis and few treatment options. With a nearly equivalent number of deaths (466,000) and reported cases (496,000) in 2020 (in percentage terms - 93.9% deaths), pancreatic cancer ranks in the seventh position in terms of cancer-related deaths in both males and females [23]. Projections indicate that pancreatic cancer is expected to overtake breast cancer and emerge as the third most common cause of cancer-related mortality in 28 European nations by 2025, owing to stable incidence rates compared to the declining numbers seen in breast cancer [24]. The aggressiveness of the malignancy makes it more difficult at later stages, which reduces the effectiveness of traditional treatment approaches. This difficulty in late-stage highlights even more how urgently novel therapeutic approaches are required. Moreover, the five-year survival rate for AdPC remains remarkably low, which emphasizes the urgent need for new and efficient treatment approaches [25].

AdPC treatment procedures typically involve chemotherapy. Combinations such as GEM with nab-paclitaxel or S-1/FOLFIRINOX (a combination of fluorouracil, leucovorin, irinotecan, and oxaliplatin) are frequently used in these protocols [26]. Although these regimens may provide patients with short-term relief, they often do not result in long-term survival and pose a significant risk of long-term side effects [27]. Surgery, a crucial early step, is less feasible in AdPC patients because of the tumors' extensive metastasis and penetration into critical structures [28]. Radiation therapy can be palliative, but its effect on OS is still limited [29]. Immunotherapies (checkpoint inhibitors, such as anti-PD-1 and anti-CTLA-4 antibodies), a novel strategy that uses the body's immune system to target and destroy cancer cells, have shown effectiveness in treating a variety of cancers but face certain challenges, such as limited efficacy of immune checkpoint inhibitors, an immunosuppressive tumor microenvironment, and a low mutational burden [30, 31].

Cancer Vaccines and WT1

The urgent need for more effective treatment modalities shifted the focus of research towards cancer vaccines. William Coley's discovery of tumor regression after bacterial infections in the late 19th century is credited with the development of cancer vaccinations [32]. However, it wasn't until the second half of the 20th century that scientists began to methodically investigate the possibility of using the immune system to specifically target cancer cells. The invention of the Bacillus Calmette-Guérin (BCG) vaccine for bladder cancer and the introduction of cytokine-based therapy are significant turning points in this endeavor [33]. The development of cancer vaccines gained momentum when it became possible to understand tumor antigens, which are proteins expressed on the surface of cancer cells that can be targeted by the immune system. The initial attempts were focused on using TAAs to trigger an immune response. However, a reevaluation of these initial attempts was necessitated by the difficulties presented by TAAs' heterogeneity and the corresponding risk of autoimmunity [34].

A new paradigm in research was brought about by the discovery of cancer genomics, which made it possible to find neoantigens, or distinct antigens arising from somatic mutations in cancer cells. Because neoantigens are unique to each patient's tumor, this tailored approach has a great deal of potential to reduce the possibility of off-target effects [35, 36]. The focus on the WT1 antigen is one of the noteworthy developments in the development of cancer vaccines [11]. The WT1 gene, which was first discovered in Wilms' tumor, produces a transcription factor that is essential for regular cellular activity, but it is overexpressed in a number of cancers, such as leukemia, ovarian, and pancreatic cancers. The characteristic trait of high expression levels in cancer cells and low presence in normal tissues is the rationale for the use of the WT1 vaccination and makes the WT1 an appealing target for better outcomes [11].

Typically, WT1 vaccines use peptide fragments obtained from the WT1 protein. These peptides stimulate the immune system by acting as immunogenic factors. Dendritic cells (DCs) and other antigen-presenting cells (APCs) are essential for ensnaring, digesting, and presenting these WT1 peptides to T cells, especially CTLs [8]. When APCs present WT1 peptides, CTLs become primed and activated to identify cells that exhibit the WT1 protein. As an essential part of the adaptive immune system, CTLs identify cells with WT1 protein as abnormal or foreign and carry out their effector roles by selectively attacking these cells [37]. These effector functions include the production of cytotoxic granules that contain granzymes and perforin, causing the target cells to undergo apoptosis. Furthermore, CTLs can induce the expression of death receptors on the surface of cancer cells, which further encourages apoptosis [38]. WT1 vaccinations elicit an immune response that attempts to create immunological memory in addition to their immediate cytotoxic effects. Following their first interaction with WT1-expressing cancer cells, memory T cells (both central and effector memory T cells) continue to exist. This memory response adds to the therapeutic effect's persistence and offers long-term protection against cancer recurrence [39].

Challenges in Using Cancer Vaccines and Future Perspectives

One significant obstacle in the field of WT1 expression is its heterogeneity across various cancer types and even within individual tumors [40]. Personalized strategies and careful patient selection are required to address this diversity, which further adds significant logistical and clinical implementation issues. The tumor microenvironment's immunosuppressive nature adds to the complexity and represents another significant challenge for WT1 vaccines. In order to evade immune surveillance and undermine the benefits of immunotherapy, tumors employ a variety of strategies, such as the overexpression of immunological checkpoint molecules. To address this difficulty, research is being conducted on the possibility of combining immune checkpoint inhibitors and WT1 vaccinations to reduce the immunosuppressive environment and improve treatment effectiveness [41].

Maximizing the effectiveness of WT1 vaccinations requires figuring out the best vaccine formulations and delivery regimens. Due to the intricacy of the immune response, the potential for immunological tolerance, and differences in patient responses, a thorough clinical evaluation is necessary to develop standardized protocols [42]. Adjuvants are essential for enhancing a vaccine's immunogenicity. It remains a challenge to find appropriate adjuvants that can successfully boost the immune response to WT1 vaccinations. Toll-like receptor agonists and other adjuvant strategies are being explored to augment the potency of WT1 vaccines [43]. The future of WT1 vaccinations will be determined by novel research avenues addressing current challenges. Cutting-edge technology such as machine learning and artificial intelligence could be crucial in predicting the best methods for producing vaccines and customizing treatment plans [44]. RNA-based vaccines, leveraging the advantages of nucleic acid technology, represent a promising avenue for the development of next-generation WT1 vaccines. These vaccinations may offer a more flexible and effective means of stimulating the immune system to target antigens unique to cancer [45].

## Conclusions

Despite being confronted with significant challenges, ongoing research into WT1 vaccines offers a promising future for cancer treatment, especially when it comes to treating AdPC, which calls for innovative approaches beyond current limitations. Targeting the overexpressed WT1 strategically highlights a prospective option for tailored therapies. Preliminary data suggest that WT1 vaccinations improve OS in individuals with AdPC, albeit the field is still in its infancy. Positive outcomes may emerge as research continues and more data becomes accessible, which could open the door for the use of WT1 vaccinations in standard care. Such an integration would signify a noteworthy advancement in the evolution of treatment strategies for pancreatic cancer patients.
